# Natural Deep Eutectic Solvents (NADES) to Enhance Berberine Absorption: An In Vivo Pharmacokinetic Study

**DOI:** 10.3390/molecules22111921

**Published:** 2017-11-08

**Authors:** Stefania Sut, Marta Faggian, Valeria Baldan, Gabriele Poloniato, Ignazio Castagliuolo, Iztok Grabnar, Beatrice Perissutti, Paola Brun, Filippo Maggi, Dario Voinovich, Gregorio Peron, Stefano Dall’Acqua

**Affiliations:** 1 Department of Pharmaceutical and Pharmacological Sciences University of Padova, via Marzolo 5, 35121 Padova, Italy; stefania.sut@unipd.it (S.S.); valeria.baldan@phd.unipd.it (V.B.); poloniato.gabriele@gmail.com (G.Pol.); gregorio.peron@phd.unipd.it (G.Per.); 2 Unir&d, Nutraceutical lab, via Tommaseo, 35100 Padova, Italy; nutraceutica@unired.it; 3 Department of Molecular Medicine University of Padova, via Gabelli, 35121 Padova, Italy; ignazio.castagliuolo@unipd.it (I.C.); paola.brun1@unipd.it (P.B.); 4 Faculty of Pharmacy, University of Ljubljana, Askerceva Cesta 7, SI-1000 Ljubljana, Slovenia; iztok.grabnar@ffa.uni-lj.si; 5 Department of Chemical and Pharmaceutical Sciences, University of Trieste, P.le Europa 1, 34127 Trieste, Italy; bperissutti@units.it (B.P.); vojnovic@units.it (D.V.); 6 School of Pharmacy, University of Camerino, Via Sant’Agostino 1, I-62032 Camerino, Italy; filippo.maggi@unicam.it

**Keywords:** berberine, NADES, HPLC-MS/MS, pharmacokinetic, in vivo

## Abstract

In the present study results related to the in vivo administration of Natural Deep Eutectic Solvents (NADES)-solubilized berberine are reported for the first time. NADES are mixtures of small natural compounds having a melting point significantly lower than that of any individual component. Such solvents have gained much attention of the scientific community in the green chemistry area, being considered useful alternatives to common organic solvents. NADES can be used also as administration vehicles, and this can be attractive for nutraceutical products when eutectics are formed with food grade ingredients. In this work, different NADES were prepared using mainly food grade constituents and were tested as solvents for the alkaloid berberine. Three selected NADES/berberine solutions and an aqueous suspension were orally administered to mice with in dose of 50 mg/Kg. Blood levels of berberine were measured by a LC-MS/MS method. The pharmacokinetic analysis revealed a 2–20 fold increase in blood concentration of NADES/berberine with significant changes in pharmacokinetic profile. Natural Deep Eutectic Solvents may thus be considered attractive solubilizing agents and may also play a role in the increase of absorption of poorly bioavailable natural products such as berberine.

## 1. Introduction

Berberine is a quaternary benzylisoquinoline alkaloid with a very ancient use in the traditional medicine of different countries, especially in China. This compound occurs in the root, rhizome and stem bark of many medicinally important plant species such as *Berberis* spp., *Coptis* spp., *Hydrastis* spp. [1].

Berberine has been used orally in China since ancient times to treat diarrhea [1,2]. This alkaloid also has a very long history of use in Ayurvedic and Chinese medicine as an antimicrobial, antiprotozoal, antidiarrhoic and antitrachoma agent [1,2,3]. In recent years, berberine has been studied for its antihypertensive, antiarrhythmic, antihyperglycemic, anticancer, antidepressant, anxiolytic, neuroprotective, anti-inflammatory, analgesic, hypolipidemic, nephron and hepatoprotective properties [4,5,6]. Other works have evidenced the antimicrobial properties of the compound against methicillin-resistant *Staphylococcus aureus* (MRSA) [6] and multidrug resistant *Escherichia coli*, antiviral effects against H1NI influenza A and activity as an inhibitor of HIV protease and HIV-I reverse transcriptase [1].

It is worth noting that this compound has also recently attracted public attention due to its cholesterol-lowering properties. Kong et al. demonstrated the ability of the alkaloid to decrease plasma cholesterol, via a mechanism related to the increase of LDL receptors [2]. Furthermore berberine is also considered potentially useful for its anti-inflammatory activity and ability to decrease free radical levels, helping the protection of the endothelial function [3,5,7]. Berberine has been studied for its potential efficacy and safety in the treatment of type 2 diabetes mellitus patients and authors have indicated that this alkaloid is a potent oral hypoglycemic agent with beneficial effects on lipid metabolism [2,3,5].

Despite its therapeutic potential, pharmacokinetic studies have indicated that berberine is poorly absorbed and that after oral administration it rapidly undergoes extensive metabolism, forming the compounds berberrubine, thalifendine, demethylenberberine, jatrorrhizine, so that its plasma concentration is extremely low [5,7,8,9]. Based on these data the oral bioavailability is considered poor [1,4,6,8,9]. Spinozzi and colleagues [8] reported that, after a single oral dose of 500 mg of berberine hydrochloride in human subjects, plasma levels of berberine, demethylberberine and jatrorrhizine were lower than 0.1 nM, showing a pharmacokinetic profile with a plateau after one hour for berberine and demethylberberine, and after 2 h for jatrorrhizine, which persisted up to 24 h. Other authors reported that oral administration of berberine chloride resulted in very low plasma levels of berberine, which is rapidly transferred to the liver and excreted with bile (mainly as thalifendine), in urine (mainly as thalifendine and berberrubine) and in feces (mainly as such) [9]. In rats, after a single oral administration of berberine (200 mg/kg) the total recovery of native compounds was 22.83%, whereas those of its metabolites were 22.74, 0.0939 and 9 × 10^−6^% in feces, urine and bile, respectively [1]. In a recent review the low oral bioavailability of berberine was attributed mainly to its poor absorption and the first-pass effect in the intestine and in the liver [9] but also to self-aggregation of the compound in gastrointestinal fluids, to its poor permeability, P-glycoprotein (P-gp)-mediated efflux, and hepatobiliary re-excretion [9].

In the recent years, other groups have worked in the improvement of berberine bioavailability [9,10,11,12,13,14,15,16]. Anionic surfactants, such as sodium caprate or sodium deoxycholate have been used as absorption enhancers. Self-emulsifying nanoparticles with sodium caprate showed a 3-fold increase of in vitro membrane permeation and a 4-fold increase of in situ intestinal perfusion, resulting in a 5-fold increase of in vivo bioavailability compared to pure berberine administered as powder or as tablets. Chitosan was also studied as a berberine carrier, demonstrating absorption-enhancing activity for its capacity to interact with the anionic components of the glycoproteins on the epithelial cell surface, regulating tight junctions, and subsequently enhancing the drug paracellular permeability [9]. Furthermore, it was proposed that chitosan, which exerts mucoadhesive properties, can increase drug retention at the mucosal surface, thus promoting absorption [9,10,11,12,13,14,15,16]. Other approaches used the co-administration of compounds able to inhibit P-glycoprotein efflux such as silymarin and tetrandrine, or a combination of d-α-tocopheryl polyethylene glycol 1000 succinate. Using a phospholipid complex of berberine, the relative oral bioavailability in rats of berberine was 322.66% in comparison to pure berberine, due to P-gp excretion inhibition and improvement of the liposolubility of berberine. Other approaches have explored the use of lipidic drug delivery systems such as nano/microemulsions, micelles, liposomes, and solid lipid nanoparticles [9].

NADES comprising natural compounds, such as organic acids, amino acids and sugars, have been recently developed for possible application in natural product field. NADES are obtained by the complexation of a hydrogen acceptor and a hydrogen-bond donor. Such solvents are almost non-volatile under ambient conditions, are chemically and thermally stable, non-flammable, and have good solvent properties for several organic compounds [11,12,13,14]. In many cases, bioactive natural compounds like berberine possess limited solubility in water and ethanol mixtures. This can be a severe limitation in the extraction of bioactive compounds for food supplements and nutraceutical ingredients [15,17]. Any alternative ideal solvent should present a high level of safety and eco-sustainability as well as improved extraction performance [11,16,18,19]. NADES can also be considered as ‘ingredients’ in nutraceuticals or functional foods, and offer the possibility of combining various molecules, leading to the preparation of tailor-made solvents employable for pharmaceutical applications [19]. Being prepared with different ingredients, NADES can be used to solubilize or extract solutes with different properties (polarity, charge, etc.) [10,11,12,13,14,15,16].

During the course of our studies to evaluate NADES as absorption enhancers for poorly bioavailable natural products [20], we considered the possibility of using this approach to increase the bioavailability of berberine. For this purpose, several NADES were prepared and used to solubilize berberine. Three mixtures were selected based on their solubilizing properties towards berberine, their different chemical composition and possible issues related to the toxicity of NADES constituents. The selected mixtures comprised basic compounds (urea) or organic acids (malic and lactic acid), and proline. These NADES with berberine were orally administered to mice and compared to an aqueous berberine suspension. Blood concentrations were monitored by using an ad-hoc developed HPLC-MS/MS method. The comparison of the pharmacokinetics of the three different NADES was used to discuss the role of eutectic solvents as absorption enhancers.

## 2. Results 

### 2.1. Berberine Solubility in NADES 

NADES composed of urea, amino acids, sugars and choline were used to solubilize berberine. The prepared eutectic mixtures are listed in Table 1. 

Water and ethanol were used as reference solvents due to their safety and favorable use as solvents in the production for nutraceuticals and functional foods. Water was furthermore selected as a reference, due to the low berberine water solubility (2.1 mg/mL at 22 °C). The solubility of berberine in each solvent is reported in Table 1. 

On the basis of their composition the prepared NADES have been classified into six groups: i.e., the first based on urea (indicated in the table with numbers 1–2), the second on an organic acid and sugars (3), the third on organic acids and amino acids (4–14), the fourth on choline chloride (15–24), the fifth on carnitine and acetyl carnitine (25–35), and the six on lactic acid (36–38). We selected proline-based NADES because in our previous study [20] proline-based NADES were efficient in solubilizing rutin. Urea-based NADES were selected because of their favorable properties in terms of polarity and capability of creating H-bonds. Owing to the basic nature of the alkaloid berberine, we also decided to prepare a large number of NADES based on organic acids. In particular, malic acid has been previously studied for its ability to accumulate berberine into cells, suggesting an important role in solubilizing the alkaloid into vacuoles [21].

Considering the results of solubility measurements, we can observe that a large number of prepared NADES were not able to increase berberine solubility compared with water or ethanol. In fact, ten different eutectic mixtures showed solubility higher than 2 mg/mL, and many of the prepared solutions suffered instability (as indicated in Table 1) resulting in precipitation of the berberine. Our results showed that choline chloride-based NADES were not efficient in the solubilization of berberine, even though in the literature such NADES have been indicated as optimal mixtures for the extraction of isoflavones from soy [22] and solubilization of rutin [20,23].

The best solubility result (25 mg/mL) was obtained with eutectic 14, (proline–malic acid–lactic acid–water 1:0.2:0.3:0.5), followed by urea-based NADES (mixtures 1 and 2) with solubility higher than 12 mg/mL. The lactic acid-based eutectics presented solubility greater than 9 mg/mL. Due to these results and considering the possible toxicity issues deriving from their composition, the following screening was carried out. The LD_50_ of urea in mice is 11,000 mg/kg, thus we selected mixture 2 instead of 1 because it contains less urea per gram. The malic acid LD_50_ in rat is 1600 mg/kg, whereas those of lactic acid in rat and mice are 3543 and 4875 mg/kg, respectively. Thus, we selected mixture 5 instead of 6 despite the fact the solubility was 2.8 instead of 6 mg/mL.

We also observed that mixture 14, containing both lactic acid malic acids and proline with quite a large amount of water presented the best solubility properties and can be considered an example of tailor-made NADES targeted for berberine. 

Thus, based on this preliminary evaluation, in order to explore the behavior of these NADES as in vivo absorption enhancers, we selected mixture 5 (henceforth indicated as “A”) and 2 (indicated as “B”) as examples of urea and malic acid-based NADES; then mixture 14 (indicated as “C”) as the best solubilizing eutectic for the pharmacokinetic evaluation.

### 2.2. HPLC-MS/MS Method Validation

#### 2.2.1. Specificity, Linearity, LOQ and LOD

**Figure 1:**
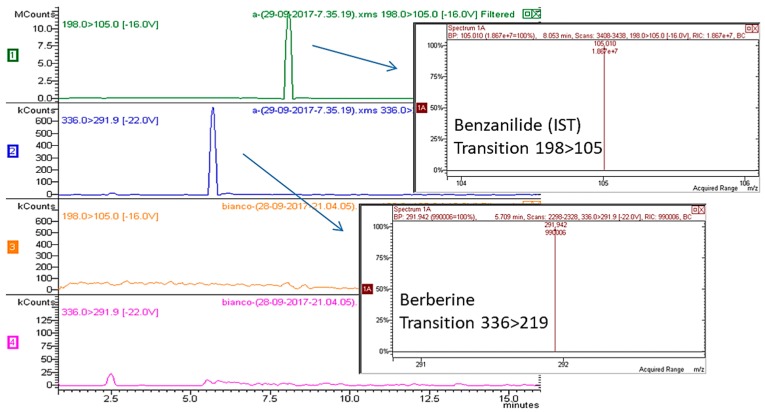
HPLC-MS/MS chromatogram of plasma spiked with ISTD and berberine and blank plasma.

Exemplificative multiple reaction monitoring (MRM) chromatograms for berberine-spiked plasma (berberine 45 ng/mL, benzanilide (the internal standard or ISTD, 100 ng/mL) are reported in Figure 1. Selected transitions for quantification were 336 → 291 for berberine and 198 → 105 for the ISTD benzanilide.

Five calibration mixtures prepared mixing different ratios of berberine/ISTD (namely, 0.008654, 0.0108, 0.04326, 0.0649, and 0.108) were used to build a calibration curve (y = area of berberine/area of ISTD; x = quantity of berberine/quantity of ISTD) that was linear and reliable over the considered calibration range. The retention times of berberine (5.7 min) and ISTD (8.0 min) and specific MS/MS transitions allowed the identification of compounds. Limits of quantification (LOQ) and detection (LOD) for berberine were 0.3 ng/mL and 0.9 ng/mL, respectively.

#### 2.2.2. Accuracy and Precision

Spiked samples were assayed for intra-day and inter-day precision and accuracy at concentrations of 0.9, 4.5, 9, 22.5 ng/mL of berberine. Data are summarized in Table 2.

### 2.3. Pharmacokinetics of Selected Eutectic Solubilised Berberin and Berberine Hydrochloride Water Suspensions in Mice

**Figure 2:**
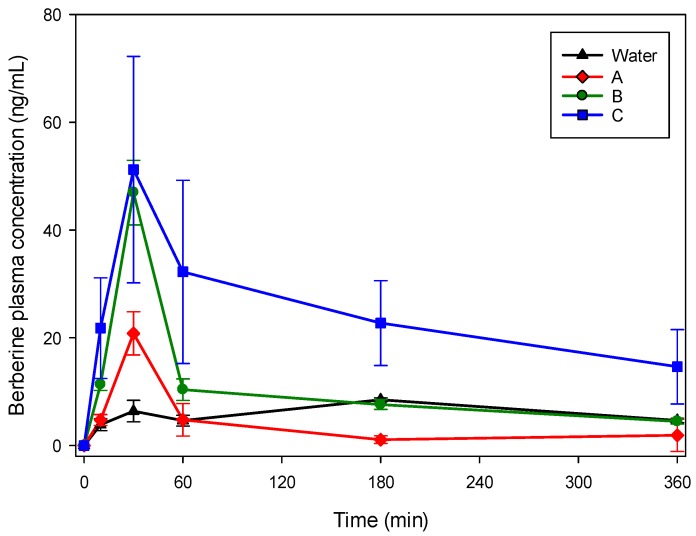
Time course (mean ± SD) of plasma concentration of berberine in Balb/c mice following oral administration of 50 mg/kg in NADES solution or suspension in water.

Eutectics formed with A, B and C were selected to evaluate their pharmacokinetic properties in mice and were compared with berberine hydrochloride water suspension. Plasma levels of berberine were determined up to 6 h after its oral administration (50 mg/kg) by gavage in mice. As reported in Table 3 and Figure 2, different plasma levels were observed. For water and the three eutectic mixtures, the maximum peak was observed after 30 min. 

The observed plasma levels of berberine administered with the three eutectic mixtures were significantly higher than that occurring in water suspension (*p* < 0.05) after 30 min. For B and C the values at 10, 60 and 180 min were also significantly different (*p* < 0.05) compared with the water suspension. The increase of the plasmatic peak at 30 min was 3.2, 7.3 and 8.0 times higher for NADES A, B and C, respectively. Table 4 summarize the non-compartmental pharmacokinetic parameters of berberine following oral administration of the same dose of berberine suspended in water or solubilized in the three considered NADES in Balb/c mice.

## 3. Discussion

Natural deep eutectic solvents can be attractive options in nutraceutical formulations and for pharmaceutical applications [15,20,24,25]. In this paper, we showed that the preparation of some NADES using mainly food grade materials, namely the eutectics A (proline–malic acid 2:1), B (proline–urea 2:1) and C (lactic acid–proline–malic acid–water) solubilized berberine better than reference solvents (water and ethanol). In particular, for the mixture C, we can explain the good performance in terms of solubility. The eutectic C contains two acids, namely malic and lactic. We selected this mixture due to the fact that malic acid was reported to form soluble berberine malate [21]. The eutectics 36–38 of the present work, containing lactic acid, were able to solubilize berberine at values of 10 mg/mL. Furthermore, the presence of a small amount of water strongly increased the solubility of berberine in the mixture C compared with the eutectics 7 and 8 (higher amounts of water) or 4–6 (without water). Our results on eutectic C are in agreement with previously published data indicating that the properties of NADES can be modulated by water due to changes in hydrogen-bonding interactions that are weakened by water dilution. The same authors also indicated that the solubility of compounds in NADES can be adjusted by changing the water content [14].

The three selected effective eutectics possess proline in their composition. On the other hand, several other tested mixtures (i.e., 3–13) containing proline were not efficient in solubilizing berberine. A further consideration is related to the presence of basic compounds such as urea, in efficiently solubilizing NADES. Also, acidic compound-based eutectics such as the lactic acid-based ones 36–38 showed good solubilization properties. The collected data show the opportunity to create specific eutectic mixtures in order to solubilize specific compounds. On the basis of the prepared eutectics we can postulate a tailor-made NADES for the berberine solubilization requires the presence of an organic acid (lactic or malic), amino acids and water. The presence of these components strongly influences the solubility properties. As a matter of fact, eutectic mixtures containing only proline and malic acid (4–5) showed better solubilization properties compared with the corresponding ones containing water (7 and 8), but mixture 14, containing 1:0.2:0.3:0.5 proline–malic acid–lactic acid–water, showed improved properties. Thus, small amounts of water are needed to increase the berberine solubilization in organic-acid-proline-based eutectics. The overall results showed a moderate ability (5- to 10-fold) of NADES to solubilize berberine with urea, lactic acid and water-containing NADES. 

Considering the pharmacokinetic analysis, the administration by gavage to mice resulted in different behavior comparing water suspension and NADES. An increase of bioavailability of berberine, especially for the eutectic C, was observed as indicated in Table 2, being the AUC four times the one observed with water suspension. The administration of NADES with dissolved berberine yielded in a significant increase of the plasma alkaloid peak at 30 min. All the prepared NADES showed similar pharmacokinetic profiles in the explored range of times, with no differences in the observed t_max_ between the urea-based and the acidic compound-based eutectics, suggesting that the increase of bioavailability observed is mainly related to the solubilization properties of the different eutectic mixtures.

## 4. Materials and Methods 

### 4.1. Chemicals

Sugars and polyols were obtained from Carlo Erba (Milan, Italy). Citric and oxalic acids were purchased from Riedel-De-Haen AG (Seelze, Germany), tartaric acid from Codex (Turin, Italy), malic acid from Carlo Erba, proline from Fagron (Bologna, Italy). Choline chloride was purchased from Sigma-Aldrich (Milan, Italy) and urea from Alfa Aesar (Karlsruhe, Germany). Glutamic acid, acetylcarnitine and carnitine were purchased from ACEF s.p.a. (Fiorenzuola D’Arda, Italy). Berberine and the internal standard benzanilide were purchased from Sigma-Aldrich. Solvents such as HPLC grade acetonitrile and methanol were purchased from Scharlau (Barcelona, Spain), and formic acid from Carlo Erba.

### 4.2. NADES Preparation

NADES have been prepared using mixtures of sugars (glucose, xylitol, sorbitol), amino acids (glutamic acid, proline), organic acids (citric, malic, oxalic and tartaric acid), and other nitrogen-containing compounds (urea, choline chloride, acetylcarnitine and carnitine). For NADES preparation the approaches described in the review of Dai et al. [11] were applied. 

### 4.3. Solubility Trials and Quantification of Solubilized Berberine in the NADES by HPLC-DAD

A precisely weighed amount of berberine was suspended in water, ethanol and in the various prepared NADES with an initial concentration of 2.5 mg/mL, then increased according to the solubility capacity of the different solvents (25 mg/mL was the highest concentration reached). Samples were placed on a magnetic stirrer (Stuart, Bibby Scientific Ltd., Stone, Staffordshire, UK) at 22 °C for 1 h, then centrifuged for 21 min at 13,000 rpm with a 5415 R centrifuge (Eppendorf, Hamburg, Germany). For quantitative measurement of solubilized berberine, a portion (100 μL) of the clear supernatant obtained after centrifugation was diluted 1:5 (*v*/*v*) in ethanol. 

For quantification, stock standard solution of berberine (100 μg/mL) was prepared dissolving the analytical standard in methanol using an ultrasonic bath. A calibration curve was obtained injecting standard solutions of berberine at different concentrations, namely 50, 25, 10, 5 and 1 μg/mL. The following calibration curve was obtained: y = 18,323–50,294 (R^2^ = 0.9997). The Limit of Quantification (LOQ) was 1 μg/mL.

For HPLC-DAD analysis, an Agilent series 1260 HPLC instrument (Agilent, Cernusco Sul Naviglio, MI, Italy) equipped with a quaternary pump, a diode-array detector, an auto sampler and a column oven compartment was used. Analyses were performed on Eclipse XDB C_8_ column (5 μm, 4.6 mm × 150 mm, Agilent). The mobile phase was (A) water–formic acid (99:1 *v*/*v*) and (B) acetonitrile. A gradient program was used as follows: 0 → 11th min: A:B (90:10) → A:B (0:100) 11 → 12th min: A:B (0:100) → A:B (0:100) 12 → 12, 10th min: A:B (0:100) → A:B (90:10) 12, 10 → 14th min: A:B (90:10) → A:B (90:10). The mobile phase flow rate was 1 mL/min and the injection volume was 10 μL. The chromatogram was recorded at 350 nm and spectral data for all peaks were obtained in the range of 190–400 nm. The retention time of berberine under these analysis conditions was 6.16 min.

### 4.4. Animals Blood Collection and Extraction

All experimental protocols involving animals were reviewed and approved by the Ethical Committee for animal Experiments of the University of Padua (CEASA; 49,571). Female, Balb/c mice (8–10 weeks old) were housed (three per cage) in polycarbonate cages and kept on a 12 h light/dark cycle. Food and water were given ad libitum. Mice, randomly divided into groups of 15 animals, one group (*n* = 15) for each NADES and berberine suspended in water. Each group received 50 mg/kg of berberine by oral gavage as water suspension (group 1) or solubilized in the selected NADES (group 2). A single blood sample was collected by cardiac puncture from each animal at 10, 30, 60, 180, and 360 min after dosing (three animals were used for each time point). The whole blood was heparinized, then three samples were obtained for each time point and each treatment group. 

### 4.5. HPLC-MS Plasma Analysis

Standard stock solutions for determination of berberine in mice blood were prepared by dissolving berberine and the ISTD benzanilide in methanol. The calibration curve was obtained mixing 500 µL of 0.8 µg/mL ISTD with different volumes (500, 300, 100, 50, and 25) of 90 ng/mL berberine standard solution in order to obtain different berberine/benzanilide quantity ratios. Mixture of ISTD and berberine were added to blank plasma samples and used for sample and calibration curve preparation. 500 µL of IS solution were added to 400 µL of whole blood (461 ng) in order to precipitate proteins. Three mL of water were added, followed by 7 mL of ethyl acetate. Samples are mixed in a vortexer and subjected to 5 min ultrasound in a bath at room temperature. The sample was then centrifuged and the clear supernatant was concentrated under vacuum at 50 °C. 200 µL of methanol were then used to dissolve the residue and used for HPLC-MS/MS measurements.

For analytical measurements, an Agilent series 1260 HPLC chromatograph equipped with a Varian Prostar 410 autosampler and coupled with a Varian 320 TQD MS spectrometer was used. The mass spectrometer was equipped with electrospray ionization (ESI) source as the interface and analysis was conducted in negative ion mode for both the analytes. Analyses were performed on a Polaris 3 C18-A 150 × 3.0 mm (Agilent Technologies). The mobile phase was (A) water–formic acid (100:1.0 *v*/*v*) and (B) acetonitrile. A gradient program was used as follows: [0 → 1th min: A:B (70:30) isocratic, 1 → 7th min: A:B (70:30) → A:B (15:85) 7 → 15th min: A:B (15:85) → A:B (15:85), and re-equilibrating time A:B (70:30) for 5 min. The mobile phase flow rate was 0.3 µL/min. The injection volume was 5 µL.

The ESI source was set in positive ionization mode. Quantification was performed using multiple reaction monitoring (MRM) with *m*/*z* 336 > 291 transition for berberine and *m*/*z* 198 > 105 transition for the ISTD. The MS parameters were capillary voltage 60 V, needle voltage 4200 V, shield voltage 600 V, collision energy 22 V, Q1 voltage 0.7 V and Q3 voltage 2.8 V, nebulizing gas pressure 50 psi and drying gas pressure 25 psi. Calibration curve using the ratio peak area berberine/peak area ISTD versus quantity berberine/quantity ISTD was *y* = 0.9701*x* + 0.0028, R^2^ = 0.9899. The limit of detection (LOD) was 0.3 ng/mL and the limit of quantification was 0.9 ng/mL.

### 4.6. Method Validation

Assay specificity was evaluated comparing the chromatograms of standard-spiked plasma with blank plasma from three different sources. Calibration curves were fitted by least square regression analysis to plot peak area ratio of berberine/ISTD relatively to the ratio of the amount of berberine/ISTD. Limit of Quantification (LOQ) was calculated as the lowest amount with a relative standard deviation <20%. Intra- and inter-day stability, extraction recovery, were measured. Precision and accuracy were evaluated using QC samples (*n* = 5) at concentrations of 0.9, 9, 22.5 and 45 ng/mL on two different days. Different plasma samples were used for intra- and inter-day stability, extraction recovery, with five replicates. 

### 4.7. Pharmacokinetic Analysis

Non-compartmental pharmacokinetic analysis was performed using WinNonlin Version 2.1 (Pharsight Corporation, Mountain View, CA, USA) software. The area under the mean plasma concentration-time curve extrapolated to infinity (AUC) was calculated using a linear-up and log-down method. Maximum concentration and the time when it was observed (C_max_ and t_max_, respectively) were reported as observed. Terminal half-life (t_1/2_) was calculated as t_1/2_ = ln2/λ_z_, where λ_z_ is the slope of the terminal phase of the plasma concentration-time curve in the semi-log plot calculated by linear regression. Mean residence time (MRT) was calculated as AUC/AUMC, where AUMC is the area under the first moment curve. Relative bioavailability F_r_ of NADES versus suspension was estimated as a ratio of AUC following the administration of NADES and AUC following the administration of the suspension.

## Figures and Tables

**Table 1:** Solubility of berberine (mean ± SD; *n* = 3) in NADES and reference solvents. The solubility in all solvents were significantly different from solubility in water (*p*-values < 0.05).

NADES Type		Composition (*w*/*w*)	Berberine Solubility at 22 °C (mg/mL)
		Water	2.10 ± 0.05
		Ethanol	2.75 ± 0.05
Urea-based	1	Proline–urea 1:1	15.0 ± 0.05
	2	Proline–urea 2:1	12.3 ± 0.07 (B)
Sugar and organic acid-based	3	Citric Acid–glucose 1:1	0.54 ± 0.02
Organic acid and amino acids-based	4	Proline–Malic Acid 1:1	2.50 ± 0.06
	5	Proline–Malic Acid 1:2	2.80 ± 0.05 (A)
	6	Proline–Malic Acid 1:3	6.00 ± 0.09
	7	Proline–Malic Acid–Water 1:2:3	1.57 ± 0.02 *
	8	Proline–Malic Acid–Water 1:2:6	0.74 ± 0.01 *
	9	Proline–Citric Acid 1:1	1.82 ± 0.04 *
	10	Proline–Citric Acid 1:2	2.45 ± 0.03 *
	11	Proline–Tartaric Acid–Citric–Acid 1:1:1	1.75 ± 0.05 *
	12	Proline–Glutamic Acid 1:1	0.45 ± 0.01
	13	Proline–Glutamic Acid 1:2	0.78 ± 0.02 *
	14	Proline–Malic Acid–Lactic Acid–Water 1:0.2:0.3:0.5	25.0 ± 0.05 (C)
Choline chloride-based	15	Proline–Choline Chloride 1:1	1.24 ± 0.02
	16	Proline–Choline Chloride 1:2	1.05 ± 0.02 *
	17	Proline–Choline Chloride 2:1	0.80 ± 0.02 *
	18	Choline Chloride–Malic Acid 1:1	0.30 ± 0.01 *
	19	Choline Chloride–Malic Acid 1:2	1.35 ± 0.06 *
	20	Choline Chloride–Malic Acid 1:3	1.56 ± 0.05 *
	21	Choline Chloride–Malic Acid 2:1	1.42 ± 0.06*
	22	Choline Chloride–Malic Acid 3:1	0.87 ± 0.05 *
	23	Choline Chloride–Citric Acid 2:1	0.75 ± 0.02 *
	24	Choline Chloride–Citric Acid 3:1	0.64 ± 0.03
	25	Carnitine–Choline Chloride 1:1	0.41 ± 0.02
Carnitine-based	26	Acetylcarnitine–Choline Chloride 1:1	0.89 ± 0.05
	27	Carnitine–Proline 1:1	1.07 ± 0.04 *
	28	Acetylcarnitine–Proline 1:1	0.85 ± 0.03 *
	29	Carnitine–Proline 1:1	1.48 ± 0.07 *
	30	Carnitine–sorbitole 1:1	0.35 ± 0.01
	31	Acetylcarnitine–sorbitole 1:1	0.27 ± 0.01
	32	Carnitine–xylitole 1:1	0.38 ± 0.01
	33	Acetylcarnitine–xylitole 1:1	0.25 ± 0.01
	34	Acetylcarnitine–proline–Lactic Acid 1:1:2	1.24 ± 0.05 *
	35	Acetylcarnitine–proline–Citric Acid 1:1:2	1.36 ± 0.04 *
	36	Lactic Acid	9.00 ± 0.05
Lactic Acid-based	37	Lactic Acid–Water–Citric Acid 1:1:0.1	10.00 ± 0.06
	38	Lactic Acid–Water–Ossalic Acid 1:1:0.1	10.00 ± 0.09

* An asterisk indicates instability of the prepared berberine in NADES solution, evidenced by precipitation or crystallization of constituents.

**Table 2:** Intra-day and inter-day precision and accuracy at different concentrations.

	Nominal Concentration ng/mL	Measured ng/mL	RSD (%)	Accuracy (%)
Intra day (*n* = 5)	0.9	0.947 ± 0.081	1.6	105.2
	4.5	4.61 ± 0.34	6.8	102.4
	9	8.92 ± 0.13	2.6	99.1
	22.5	23.2 ± 0.68	13.7	103.2
Inter day (*n* = 5)	0.9	0.934 ± 0.088	1.9	103.7
	4.5	4.56 ± 0.24	7.0	101.3
	9	8.93 ± 0.17	3.8	99.2
	22.5	23.0 ± 0.51	11.0	102.2

**Table 3:** Mean (mean ± DVST, *n* = 3) measured plasmatic concentrations of berberine after oral administration (50 mg/kg) in mice. Used eutectics are indicated with letters, namely, A: Proline Malic Acid 2:1, B: Proline Urea 2:1, C: Lactic Acid: Proline: Malic Acid: Water.

Time (Min)	Water	A	B	C
	ng/mL	ng/mL	ng/mL	ng/mL
10	3.83 ± 0.51	4.75 ± 0.49	11.33 ± 1.10	21.80 ± 2.88
30	6.40 ± 0.32	20.71 ± 3.63	46.94 ± 6.00	51.21 ± 9.20
60	4.66 ± 0.36	4.73 ± 1.81	10.36 ± 2.00	32.23 ± 8.50
180	8.50 ± 1.70	1.10 ± 0.09	7.60 ± 0.90	22.72 ± 5.00
360	4.68 ± 0.37	0.74 ± 3.00	4.50 ± 0.50	14.62 ± 6.91

**Table 4:** Non-compartmental pharmacokinetic parameters of berberine following oral administration in Balb/c mice.

	Suspension	A	B	C
t_max_ (min)	180	30	30	30
C_max_ (ng/mL)	8.5	20.8	46.9	51.2
t_1/2_ λ_z_ (min)	208	268	248	265
AUC_last_ (ng min/mL)	2226	1170	3499	8640
AUC (ng min/mL)	3630	1904	5112	14,219
AUC (%Extrapolated)	39	39	31	39
MRT (min)	371	354	315	384
F_r_ (%)	100	52	141	392
